# Classification and Detection of Breathing Patterns with Wearable Sensors and Deep Learning

**DOI:** 10.3390/s20226481

**Published:** 2020-11-13

**Authors:** Kristin McClure, Brett Erdreich, Jason H. T. Bates, Ryan S. McGinnis, Axel Masquelin, Safwan Wshah

**Affiliations:** 1 College of Engineering and Mathematical Sciences, The University of Vermont, Burlington, VT 05405, USA; Kristin.Mcclure@uvm.edu (K.M.); Ryan.McGinnis@uvm.edu (R.S.M.); Axel.Masquelin@uvm.edu (A.M.); 2 Department of Medicine, Larner College of Medicine, University of Vermont, Burlington, VT 05405, USA; berdreic@health-quest.org (B.E.); jason.h.bates@med.uvm.edu (J.H.T.B.)

**Keywords:** breathing pattern detection, breathing pattern classification, deep learning, sleep apnea obstructive, sleep apnea central, biomarkers

## Abstract

Rapid assessment of breathing patterns is important for several emergency medical situations. In this research, we developed a non-invasive breathing analysis system that automatically detects different types of breathing patterns of clinical significance. Accelerometer and gyroscopic data were collected from light-weight wireless sensors placed on the chest and abdomen of 100 normal volunteers who simulated various breathing events (central sleep apnea, coughing, obstructive sleep apnea, sighing, and yawning). We then constructed synthetic datasets by injecting annotated examples of the various patterns into segments of normal breathing. A one-dimensional convolutional neural network was implemented to detect the location of each event in each synthetic dataset and to classify it as belonging to one of the above event types. We achieved a mean F1 score of 92% for normal breathing, 87% for central sleep apnea, 72% for coughing, 51% for obstructive sleep apnea, 57% for sighing, and 63% for yawning. These results demonstrate that using deep learning to analyze chest and abdomen movement data from wearable sensors provides an unobtrusive means of monitoring the breathing pattern. This could have application in a number of critical medical situations such as detecting apneas during sleep at home and monitoring breathing events in mechanically ventilated patients in the intensive care unit.

## 1. Introduction

The cyclic variations in lung volume that take place during breathing comprise a vital sign of fundamental importance to clinical medicine. The patterns that these volume variations present can vary dramatically, from the fairly regular rhythm that characterizes health to a complete absence of breathing indicative of various life-threatening conditions. A wide range of intermediate possibilities corresponds to physiologic abnormalities such as the hypopneas associated with central respiratory depression to the rapid shallow breathing that portends the likelihood of successful weaning from mechanical ventilation [1,2]. Abnormalities in the pattern of breathing can be persistent in the case of chronic diseases, but can also develop extremely quickly in medical emergencies, so being able to diagnose such patterns quickly and reliably is often critical.

Assessing abnormalities in the breathing pattern has been part of basic medical diagnosis for centuries, but has relied on the availability of appropriately trained personnel. Many opportunities to diagnose pathologies, or intervene to save lives, have thus been lost because such personnel cannot always be present when needed. Accordingly, the reach of medical care would be expanded significantly by the ability to reliably detect and classify abnormal breathing patterns without the physical presence of medical professionals. Modern sensors and signal processing methods now make this a clear possibility.

Significant progress has been recently made in the use of wearable sensors for monitoring breathing [3]. These sensors have the potential to serve as non-invasive and cost-effective tools to capture breathing patterns. Indeed, there is growing interest in using wearable devices and machine learning models for the early detection and prevention of serious respiratory illnesses. Most prior studies have been designed to classify breathing events as being either normal or abnormal using binary classification techniques. The typical approach is to use a sliding window of single or multiple lengths to detect events [4,5,6,7]. The drawback of this approach, however, is the computational complexity and complicated implementation that varies based on event length, event overlap, and event type.

The field of medicine probably most in need of automated methods of breathing pattern detection and classification is that concerned with sleep apnea [4,5,6]. The public health burden of sleep apnea is difficult to estimates accurately because many cases go undiagnosed, and it varies dramatically ion severity, but it is widely accepted that it affects millions of people in the US and is increasing because of the rise in the incidence of obesity [8]. The standard approach to diagnosing sleep apnea is polysomnography, where a subject sleeps in a clinic setting while instrumented with a variety of sensing devices [9]. This procedure is cumbersome for the subject, however, and often presents a challenge to normal sleep, so home sleep monitoring of a less complete signal set is an area of significant current interest.

The analysis of polysomnographic data is also an area in need of automatic methods. Traditionally, a large number of continuously recorded signals from an entire night of sleep are parsed by a skilled human observer for events indicative of upper airway obstruction of cessation in the drive to breathe. There is thus great interest in methods that can reliably take over this extremely time-consuming and labor-intensive activity [9]. Machine learning presents an obvious opportunity in this regard and indeed has been investigated with respect to the detection of arousals during sleep as reflected in polysomnographic data.

In the present study, we investigated the use of end-to-end machine learning techniques to detect and classify various breathing patterns based on the acceleration and rotation of two body points, one on the thorax and one on the abdomen. We used non-invasive light-weight wireless sensors that are virtually imperceptible to the subject, which is crucial because the breathing pattern itself can be modified substantially by the perceived presence of a measuring apparatus. We leveraged recent advances in deep learning to arrive at an algorithm that recognizes discriminatory patterns in the data, much like a human expert does, but without the need to develop explicit methods of signal processing, something that has proven difficult to do in a generally reliable fashion. We anticipate our results will have applications in a variety of medical scenarios, including sleep apnea, long-term mechanical ventilation, and infant monitoring.

The rest of this paper is organized as follows: Section 2 discusses related work. Section 3 introduces the data collection approach, dataset, data pre-processing, and experiments. Section 4 and Section 5 provide the results and discussion, respectively.

## 2. Related Works

Using wearable devices to monitor breathing is currently an active area of research that is focused mainly on sleep apnea [4,5,6,7,10] and respiratory rate detection [11,12]. Sleep apnea studies, however, typically only classify sensor signals as representing either apnea or non-apnea, without specifying where in time the events begin and end. One study, for example, achieved an 88% accuracy rate in classifying 32 patients as being impacted by apnea based on an electrocardiogram signal [4], while another achieved a 79–82% accuracy of apnea classification using a neural network with unspecified generic sensors [5]. A neural network binary apnea classifier was also used on 10 patients fitted with an accelerometer on the arm and chest and yielded a mean absolute error of less than 2.6 breaths/min [6]. A proof-of-concept study involving four patients explored the use of smartphones to detect sleep apnea [7], although no metrics of success were given. A number of other studies have described machine learning algorithms for the classification of sleep apnea events without the use of data from wearable devices.

The present study is an advance on this prior work as it combines the use of light-weight wireless sensors with a new deep learning method that not only classifies breathing events but also detects where they start and end in the data time series.

## 3. Methods

### 3.1. Wearable Sensor Data Collection

We collected data from 100 healthy volunteers who gave written informed consent in a protocol that was approved by the Institutional Review Board of the University of Vermont. Exclusion criteria included a history of lung disease and/or smoking, and an active respiratory tract infection. Subject demographics are given in Table 1. The study subjects were younger on average than a typical population with sleep apnea [13,14].

Under the supervision of a physician, comfortable skin-worn inertial sensors (BioStamp nPoint, MC10, Inc., Lexington, MA, USA), set to record tri-axial accelerometer and angular rate gyroscope data at a rate of 125 Hz, were attached to the chest and upper abdomen of each subject using a double-sided biocompatible adhesive. The physician instructed each patient to perform the following breathing patterns:

1. Five min of normal breathing.
2. Thirty-seconds at functional residual capacity without any breathing movements to simulate central sleep apnea.
3. Five simulated yawns.
4. Five simulated coughs.
5. Five simulated sighs.
6. Five Mueller maneuvers (attempting to breathe deeply with mouth closed and nose pinched) to simulated obstructive sleep apnea.

Subjects 1–13 performed the five repetitions of the above-simulated events within a single recording epoch, which proved useful for validating the method we developed for generating synthetic data sets (see below). Subjects 14–100 performed the breathing events within separate recording epochs to make it easier to isolate the events for subsequent analysis. The order in which the various breathing events were performed was randomly selected for each subject.

**Figure 1:**
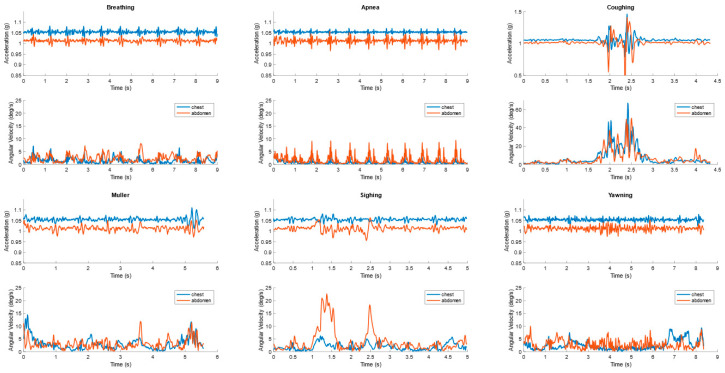
An example of the six breathing patterns captured from the accelerometer and gyroscope sensors on the chest and abdomen.

Data were annotated for each breathing event by the physician supervising data collection. Each data recording contained 12 channels corresponding to accelerometer and gyroscope readings along each of three orthogonal axes for each of the two wearable sensors. Figure 1 shows an example of the six breathing patterns (Normal breathing, Apnea, coughing, Muller, Sighing, and Yawning) captured from the two wearable sensors. The curves are the vector magnitude of acceleration and angular velocity at the chest and abdomen. For the normal breathing and simulated central sleep apnea events, the first nine seconds of the data are shown.

**Figure 2:**
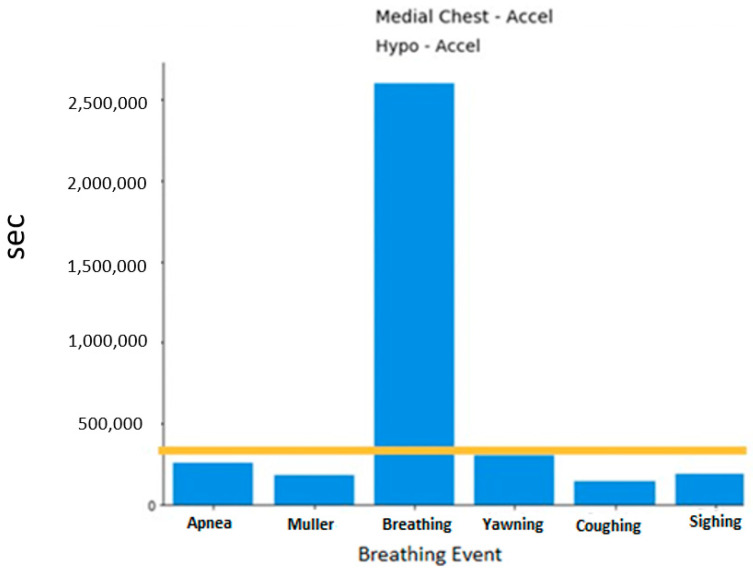
Histogram of breathing events by different sensor locations.

A histogram of the breathing events can be viewed in Figure 2. As expected, the normal breathing events dominate as they were collected for 5 min. Each segment of data for training and testing uses normal breathing as the basis. This will be further discussed in the following section.

### 3.2. Data Preprocessing

The intention was to have this one-dimensional convolution neural network (1D-CNN) learn to recognize the various breathing events directly from the raw data, so no cleaning or filtering was applied. For classification, seven-second windows of data were extracted from each breathing event completed by each subject.

For detection, we randomly selected 40,000 segments of normal breathing data from the entire subject dataset. Each segment was 30 s in length (3750 data points per segment), for a total of 1.2 million seconds of data. Each consecutive second in each segment was assigned a label denoting the kind of breathing event it contained (initially all normal breathing). We then replaced selected portions of each segment with simulated events representing either central sleep apnea (CSA), coughing, obstructive sleep apnea (OSA), sighing, or yawning that had been isolated from the subject dataset. The 30 labels for each segment were adjusted accordingly. For segments that had one breathing event injected, this occurred at any location within the 30-s segment. For segments that had two breathing events injected, the first event was injected randomly in the first 15 s, while the second event was injected randomly within the second 15 s, thus ensuring that the two events did not overlap.

The injected events were randomly drawn from the pools of CSA, coughing, OSA, sighing, and yawning events. If a segment was injected with only a single CSA event, the event duration was randomly set to be 10, 20, or 23 s, consistent with the clinical definition of central sleep apnea [15]. If one of the other breathing events was injected, the entire event was included (none of these other events exceeded 14 s). For segments that had two events injected, if one of these was CSA, its duration was randomly selected to be either 10, 11, or 13 s.

**Figure 3:**
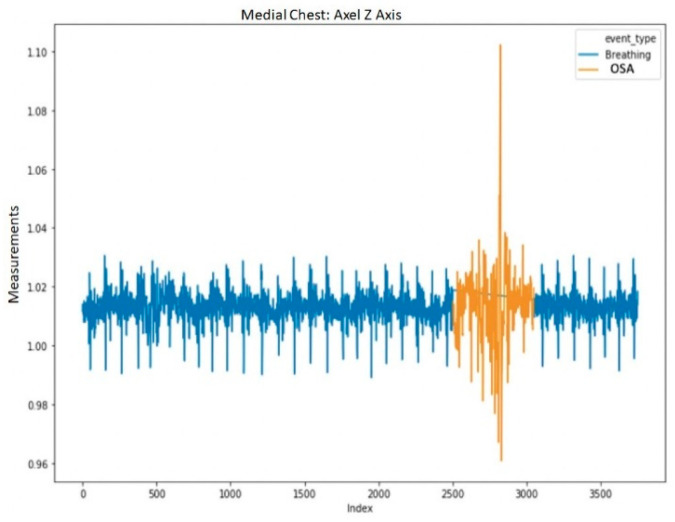
Segment example of the medial chest, accelerometer *Z*-axis (roughly normal to the surface of the chest) with the inserted Obstructive Sleep Apnea event highlighted.

**Figure 4:**
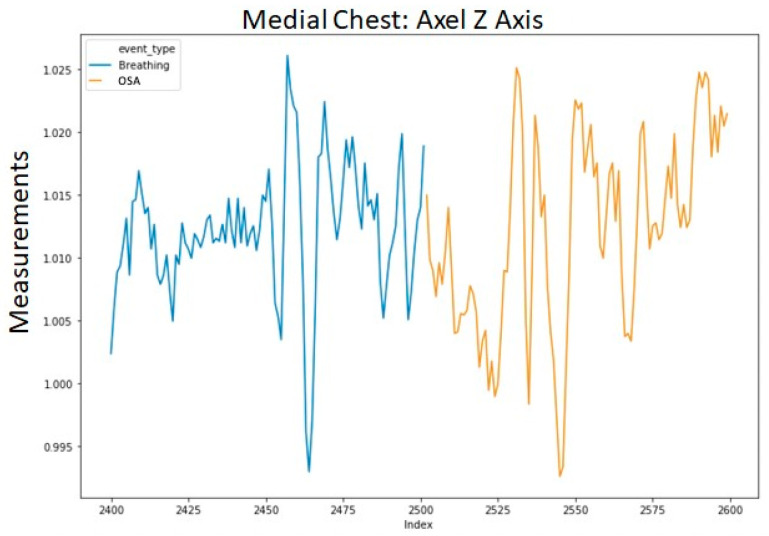
Zoomed in segment example of the medial chest, accelerometer *Z*-axis (roughly normal to the surface of the chest) with the inserted Obstructive Sleep Apnea event highlighted to demonstrate the relatively smooth transition.

To avoid excessively sharp transitions between normal breathing and each injected event, we replaced the first and last data points of each injected segment with their respective averages with the adjacent point from normal breathing. The averaged data points retained the label of the injected event. This procedure was applied to each of the 12 channels in the data. An example of a segment with a single injected event (OSA) is shown in Figure 3. The transition into the event in Figure 3 is shown in a more granular view in Figure 4.

For all the experiments described in the next sections, 60% of the 40,000 segments were assigned for the training set, 20% for the validation set, and 20% for the testing set.

We performed our data analysis both on the entire dataset and on segments containing only the accelerometer readings as accelerometers require less electrical power than gyroscopes [16]. However, the latter provided much less robust results. In fact, we saw a drop in performance of approximately 30% when we used either the accelerometer or gyroscope channels alone. Accordingly, the model discussed below utilizes all 12 raw channels in each segment.

## 4. Experiments

We first implemented a deep learning classification system. The classifier was implemented in both binary and multi-event forms. The more challenging task of classifying events as being any of the five simulated breathing patterns (OSA, CSA, coughing, sighing, and yawning) was performed by the multi-event classifier.

We then implemented a deep learning detection system that assigned an event designation (normal breathing, OSA, CSA, coughing, sighing, and yawning) to each consecutive second of a data segment. This system could potentially be used for detecting and classifying breathing events in a clinical setting.

To achieve both classification and detection, we implemented 1D-CNN using the python deep learning framework Keras [17], along with the Scikit-learn open-source library for evaluation methods [18]. We used the Vermont Advanced Computing Core (VACC) to train and evaluate our algorithms. We fitted our deep learning using a single NVIDIA GPU Tesla V100s with 32 GB RAM.

### 4.1. Binary Classification

**Figure 5:**
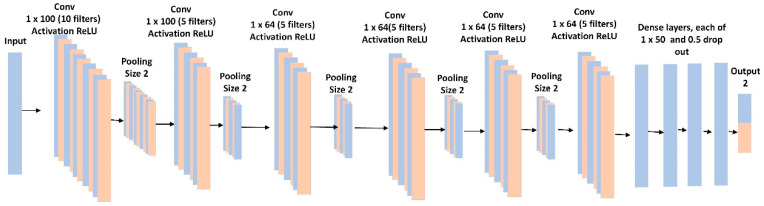
1-D convolutional neural network for binary and multi-event classification.

The binary classification was performed using a 1D-CNN similar to an AlexNet [19], in which there were 6 convolutional layers and 4 fully connected layers (Figure 5). The model included batch normalization and max-pooling after each convolutional layer. To control for overfitting, we used L2 regularization of 0.1, and a dropout rate of 50% for each fully connected layer. The last layer provided the binary classification and used a sigmoid activation function. We used a binary cross-entropy loss function with the RMSprop optimizer and a learning rate of 0.001. We optimized batch size, learning rates, loss function, and time slide windows using the random grid search described in [20].

We balanced the dataset across the breathing event classes by applying a clip level to ensure a similar number of segments for each class. This gave 369 segments of each event class. All 12 raw data channels were used, and no feature engineering was applied. The input to the 1D-CNN consisted of 7 s for each event at 125 Hz (875-time steps) multiplied by 12 raw channels, for a total of 10,500 features.

### 4.2. Multi-Event Classification

For the multi-event classification experiments we used a similar 1D-CNN as for the binary classification experiments described above, but with the following exceptions. The last layer included the six classification classes with a softmax activation. We used a categorical cross-entropy loss function with the RMSprop optimizer. To control for overfitting we used L2 regularization of 0.001. We optimized batch size, learning rates, loss function, and time slide windows using the random grid search described in [20]. The training time for both binary and multi-classification was approximately 12 h.

### 4.3. Detection

**Figure 6:**
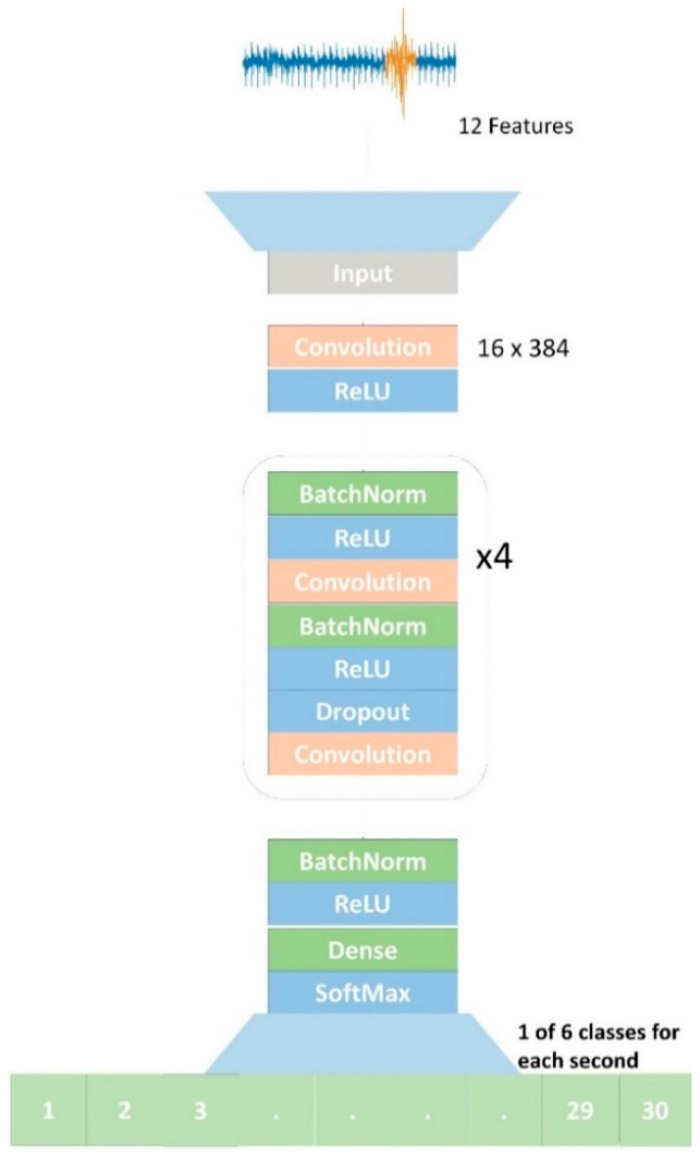
Deep neural architecture for the detection model.

The detection model illustrated in Figure 6 received inputs from the 12 raw features in the 30 s data segments and predicted a label for each second within the segment. The model consisted of a 34-layer Convolution Neural Network (CNN) beginning with a convolutional layer followed by batch normalization with a ReLu (rectified linear) activation function. We used residual blocks to provide the benefit of relaying information across the entire network [21]. There were 4 residual blocks each containing 2 convolutional layers. The model concluded with a fully connected layer having a time dimensional component and a softmax.

The input to the detector consisted of the 30 s data segments each with 12 raw features sampled at 125 Hz, for a total of 3750 input data points. The detector output consisted of a label (normal breathing, CSA, coughing, OSA, sighing, and yawning) for each second of a segment containing one of the six classes of breathing events. For testing, there were 26.1K s of CSA, 123.1K s of normal breathing, 7.6K s of coughing, 8.9K s of OSA, 8.8K s of sighing, and 15.4K s of yawning.

We used the Adam optimizer with a learning rate of 0.001, which then was reduced by a factor of 10 if the network’s progress slowed [22]. The loss function was categorical cross-entropy. For the residual blocks, the model started with 64 filters and doubled each residual block resulting in the final residual block having 512 filters. For the first convolutional layer, we used a filter length of 384, which is considered a relatively large filter length (or kernel size) [23] but allowed the model to view 3 s of data at a single instance. While this made the model very large, resulting in over 201 million parameters, we found a large kernel size was critical for detecting the events correctly. The training time for the detection task was approximately 36 h on one GPU. To speed up the hyper-parameter search process, we parallelized the computation by running multiple experiments on different GPUs.

## 5. Results

### 5.1. Binary Classification Model

**Figure 7:**
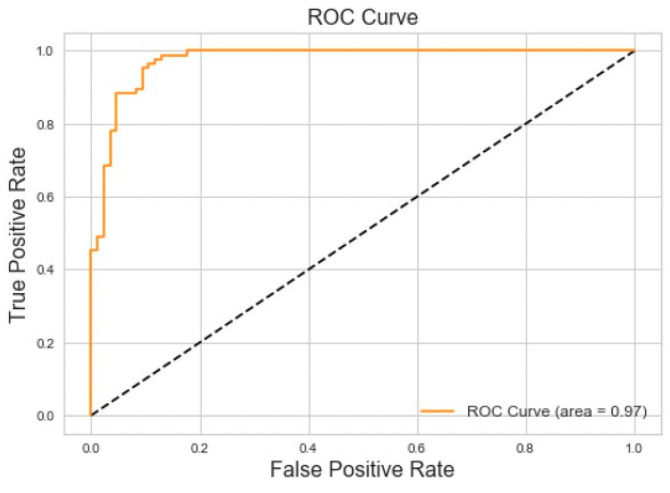
Receiver operating characteristic (ROC) curve for central sleep apnea vs. normal breathing.

We evaluated various time window parameters for the binary classification model and determined that 7 s was the optimal window length because it captured the entirety of each breathing event. Applied to the balanced dataset, the binary classifier achieved 95% accuracy and 95% area under the ROC curve for Central Sleep Apnea versus normal breathing on the testing dataset. The results of this experiment can be seen in Figure 7.

**Figure 8:**
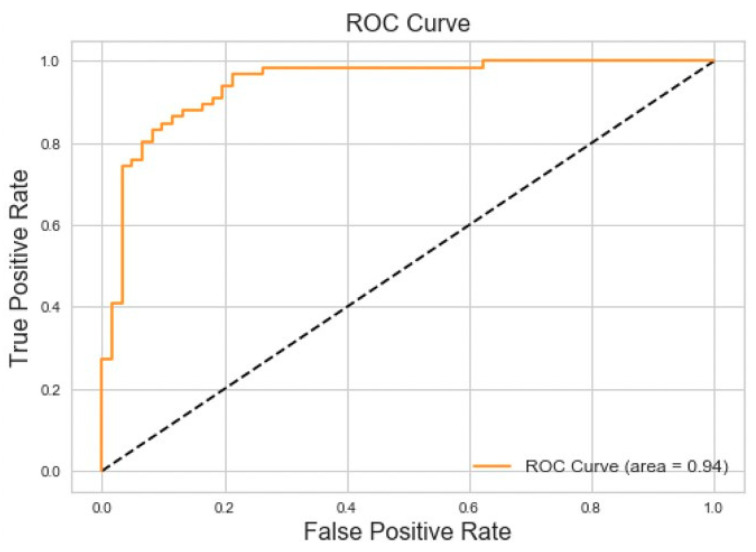
ROC curve for obstructive sleep apnea vs. normal breathing.

Binary classification for Obstructive Sleep Apnea versus Normal Breathing achieved 86% accuracy and 94% area under the Receiver Operating Characteristic (ROC) curve for the testing dataset. The results of this experiment can be seen in Figure 8.

### 5.2. Multi-Event Classification Model

**Figure 9:**
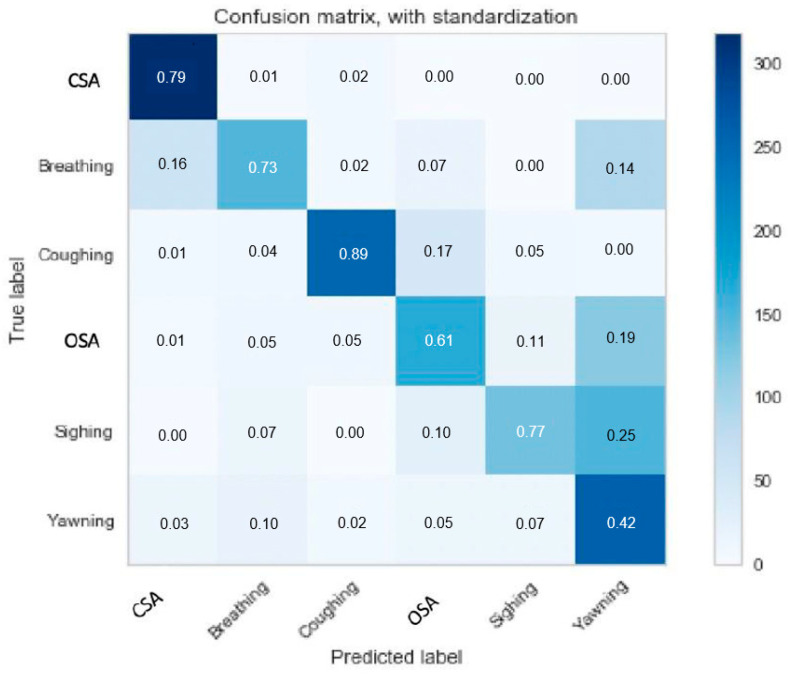
Confusion matrix for multi-event classification.

Multi-event classification achieved F1 scores of 87% for CSA, 56% for normal breathing, 83% for coughing, 55% for OSA, 53% for sighing, and 55% for yawning on the testing dataset as showed in Table 2. The model had a limited ability to differentiate between sighing and yawning, which is not surprising given the similarity of the two events. Introducing sighing and yawning also made it challenging for the model to accurately classify OSA. The results of the multi-event classification experiment are shown in Figure 9.

**Figure 10:**
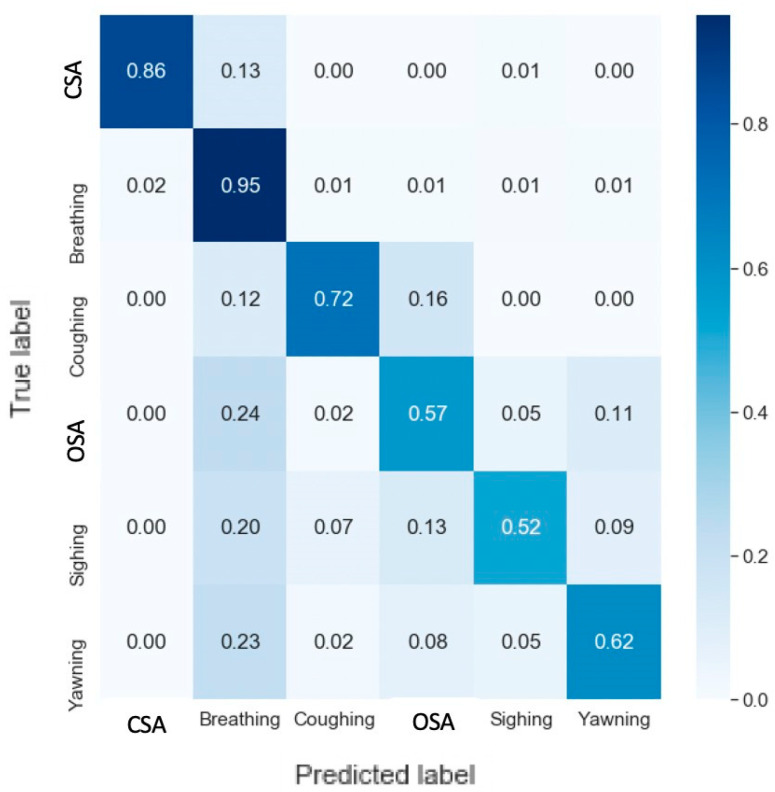
Confusion matrix of the detection model test results.

The confusion matrix for the testing of the detection model is shown in Figure 10. CSA, normal breathing, and coughing all had high F1-scores (86%, 95%, and 72%, respectively). When CSA was predicted incorrectly, it was identified as normal breathing 13% of the time, while OSA, sighing, and yawning were incorrectly identified as normal breathing 20–24% of the time. OSA was also incorrectly identified as yawning 11%. Incorrect detection tended to occur at locations in the data segments that were transitioning between the background of normal breathing and the injected events. For example, if there was 4 s of normal followed by a 10 s CSA event followed again by 16 s of normal breathing, the model identified 5 s of normal breathing followed by 10 s of CSA followed by 15 s of normal breathing, demonstrating a 1-s lag in detection relative to the data. Table 3 shows the full report of the detection model applied to the testing dataset.

## 6. Discussion

CNN’s have recently proven remarkably adept at performing classification and recognition tasks previously thought to be the exclusive domain of the human observer. While 2-dimensional CNNs are often used for image recognition, 1-dimensional CNN’s are proving very useful for the analysis of sensor data [24]. The first convolutional layers in a CNN learn local patterns (such as edges) while the subsequent layers learn more abstract and complex patterns [25]. At a high level, a CNN takes input data (in this case, time series of accelerometer and gyroscope readings), assigns importance (learnable weights) to aspects of the data to differentiate them, and then provides a classification output (in this case, a designated breathing event).

The binary classification results we obtained for OSA and CSA versus normal breathing (Figure 6 and Figure 7) are comparable to those obtained with other biomedical signals. For example, PhysioNet Apnea-ECG data gave a classification accuracy of 88% when applied to a neural network [4]. Other studies that incorporated wearable sensors and neural networks for sleep apnea monitoring reported accuracies no higher than 93% [5,6,7,26,27,28]. This suggests that the classification system developed in the present study could be a viable option for in-home sleep studies.

The detection model was able to accurately separate breathing events from normal breathing with a temporal resolution of 1 s (Figure 10), whereas the multi-event classification model achieved similar accuracy on the much less challenging task of identifying only 1 or 2 events per data segment (Figure 9). We suspect this reflects the greater amount of training data used with the detection model and the longer data window it was presented with. The fine-grained second-by-second labeling in the training set provided more detailed temporal information to the detection model. The confusion between the non-normal breathing events was similar for the detection and the multi-event classification models, with CSA and coughing being the easiest to distinguish while sighing and yawning were the most challenging to distinguish due to their similarities.

An interesting question that always arises in applications of deep learning relates to the specific features in the data that the algorithms settle upon as being key to their decision making. Answering such questions can be challenging because these features are not represented explicitly in algorithm behavior. Indeed, there is no guarantee that the information extracted from training data by a deep learning algorithm would even be readily recognizable as a feature in the conventional sense. We are thus not sure of the basis upon which the algorithms in the current study made their decisions, although we might make some reasonable speculations. In particular, one of the distinguishing features of OSA is that abdominal and thoracic movements become out of phase as the respiratory muscles compete with each other for a volume of thoracic gas that remains fixed because the upper airway is occluded. Robles-Rubio et al. [26] used the phase of thoracic versus abdominal movements, along with 5 other waveform-derived features, to classify breathing patterns in infants using k-means classifiers. These investigators found pattern-specific confusion levels of roughly 20%, which is similar to the levels of confusion we found for the detection of breathing versus OSA, sighing, and yawning (Figure 10). It is thus conceivable that our deep learning algorithms identified similar features as relevant to its own decision making. In this regard, visualizing the responses of the CNN filters might provide insight into the features that were identified as being most important for breath classification, although the size of the first filtering layer in our CNN was 100 for classification and 384 for detection, so the features could be quite extended in time and thus difficult to interpret.

The deep learning architectures developed in this study are derived from state-of-the-art approaches [28]. In particular, the very large number of hyper-parameters in the models we developed required that we optimize their designs using the random search grid algorithm described in [20]. Specifically, we determined model performance using multiple values for each hyper-parameter to find the optimal combination. This procedure was computationally expensive, but it resulted in a much-improved performance and demonstrates the importance of investing sufficient effort up-front in a machine learning architecture.

Nevertheless, our study has a number of limitations. CNNs can learn patterns that are translation invariant. Since we did not take subject movement or changes in position into account, our CNN’s were not trained to deal with these eventualities. While the detection model proved reliable at detecting CSA with second-by-second resolution, even though simulating CSA by breath hold was a somewhat difficult maneuver for subjects to perform consistently, the detection model had a more limited ability to detect OSA. The latter is an even more challenging event for conscious normal subjects to simulate. Testing of the performance of the detection model after it has been trained on data collected from actual sleep apnea patients is therefore an important area for further investigation.

The fact that our 1D-CNN miss-classified some cases of OSA, yawning, and sighing does not come as a surprise given the similarities between these events; many subjects performed these events in much the same fashion. More extensive subject training may have provided better discrimination, but this would have extended the study duration considerably and thus would have limited subject recruitment to the study.

It is also important to note that the 100 patients involved in our study simulated six different breathing events in a controlled setting, and the position of each patient was consistent during the data collection process. This does not necessarily accurately mimic an in-home setting or a sleep clinic environment in which subjects may adjust their postures frequently. Furthermore, the subject demographics of the present study do not accurately represent those of patients at risk for sleep apnea OSA [13].

## 7. Conclusions

We have demonstrated that wearable sensors recording acceleration and rotation of points on the thorax, when paired with deep learning algorithms, can detect and classify different breathing patterns of medical significance. In particular, they can detect breathing events associated with sleep apnea, potentially allowing cheaper and less cumbersome approaches to non-invasive sleep monitoring at home and in the clinic compared to conventional polysomnography.

The optimized 1D-CNN we developed has 32 layers, a large kernel size, and residual blocks that together were able to assign one of six possible breathing events to each sequential second of 3-axis accelerometer data from the chest and abdomen. The next step is to apply this methodology to patients in clinical situations, most especially the sleep lab and the intensive care unit.

## Figures and Tables

**Table 1:** Patient study gender, age, weight, body mass index (BMI) breakdown.

Gender	Count	Average	Weight	BMI
Female	54	35	147	26
Male	46	31	185	27
Total	100	33	165	26

**Table 2:** Scores. After performing multiple runs with the optimized model, the average F1 score was found to be 87%.

	Precision	Recall	F1-Score	Support
Apnea	0.79	0.97	0.87	329
Breathing	0.72	0.46	0.56	326
Coughing	0.89	0.77	0.83	329
Muller	0.61	0.50	0.55	329
Sighing	0.77	0.40	0.53	329
Yawing	0.41	0.80	0.55	325

**Table 3:** Detection report of the test results.

	Precision	Recall	F1-Score	Support
CSA	0.89	0.85	0.86	26,065
Breathing	0.90	0.95	0.95	123,061
Coughing	0.75	0.71	0.72	7627
OSA	0.52	0.50	0.57	8864
Sighing	0.61	0.54	0.52	8801
Yawing	0.74	0.56	0.62	15,402
